# Dietary niacin intake in relation to depression among adults: a population-based study

**DOI:** 10.1186/s12888-023-05188-8

**Published:** 2023-09-18

**Authors:** Sheng Tian, Lanxiang Wu, Heqing Zheng, Xianhui Zhong, Mingxu Liu, Xinping Yu, Wei Wu

**Affiliations:** 1 Department of Neurology, The Second Affiliated Hospital of Nanchang University, No.1, Minde Road, East Lake District, Nanchang, 330006 People’s Republic of China; 2 Institute of Neuroscience, Nanchang University, No.1, Minde Road, East Lake District, Nanchang, 330006 People’s Republic of China

**Keywords:** Depression, Niacin, U-shaped, Cross-sectional study, NHANES

## Abstract

**Background:**

Previous studies have shown that an antioxidant diet is a protective factor against depression. However, the association between niacin, an important antioxidant consumed from the diet, and depression has received little attention. Therefore, we explored the association between niacin intake and depression through a cross-sectional analysis of the National Health and Nutrition Examination Survey (NHANES) from 2007 to 2016.

**Methods:**

Depression was measured using the Patient Health Questionnaire (PHQ-9, score ≥ 10). Niacin intake was assessed through 24-h dietary recall interviews. The relationship of niacin intake with depression among adults in US was assessed by using a weighted multiple logistic regression model with subgroup analysis. Non-linear associations were explored using restricted cubic spline models. And we used a two-piece-wise logistic regression model with smoothing to explore the threshold for association between them.

**Results:**

A total of 16,098 adults were included in this study. Compared with individuals with lowest niacin intake Q1 (≤ 15.96 mg/day), the adjusted OR values for dietary niacin intake and depression in Q2 (15.97–22.86 mg/day), Q3 (22.87–32.28 mg/day) and Q4 (≥ 32.29 mg/day), were 0.92 (95% CI: 0.70–1.20), 0.76 (95% CI: 0.56–0.99,) and 0.68 (95% CI: 0.48–0.98), respectively. The results were not modified by sex, by age and by BMI. Furthermore, the relationship between dietary niacin intake and depression exhibited a U-shaped curve (nonlinear, p < 0.001). And depression risk was lowest when dietary consumption of niacin was around 36 mg/day.

**Conclusions:**

In present study, moderate niacin intake, but not high intake, was associated with lower odds of depression suggesting a U-shaped association.

**Supplementary Information:**

The online version contains supplementary material available at 10.1186/s12888-023-05188-8.

## Background

Depression is a common psychiatric disorder worldwide, which reported with an increase of 49.86% in the number of people suffering from depression between 1990 and 2017 [1]. People with depression have a significantly lower quality of life than healthy people, and depression is recognized as a major contributor to suicide and disability [2, 3]. However, depression continues to have a serious long-term impact on people’s health and quality of life due to a lack of effective treatments and inadequate mental health resources. A recent meta-analysis suggested that depression was associated with nutrients [4], which may alleviate depressive symptoms or reduce the prevalence of depression [5, 6]. Therefore, it is crucial to explore other potential dietary nutrients associated with depression, which may aid in preventing or treating depression.

Niacin (vitamin B3) is a nutritional precursor to nicotinamide adenine dinucleotide phosphate (NADP) and nicotinamide adenine dinucleotide (NAD), which are essential cofactors for mitochondrial energy metabolism [7]. Some studies have shown that B vitamins play a vital role in the development, maintenance and function of the brain, but severe deficiencies are associated with an increased prevalence of mental disorders [8, 9]. Also, deficiency of niacin, one of the B vitamins group may reduce oxidative phosphorylation and impair mitochondrial respiration [10]. Previous research indicated that the trinity of brain energy deficit, mitochondrial dysfunction and oxidative stress might exert a role in the development of depression [11, 12]. However, there are rare studies examining the association between dietary niacin intake and depression in the general population.

Therefore, the relationship between dietary niacin intake and depression in adults was assessed with data from the NHANES to refine this study in the general population. Based on the nutritional patterns found in this population, we hypothesized that there was negative correlation between dietary consumption of niacin and depression. We also conducted subgroup analyses to assess possible effect modification of the association between dietary niacin intake and depression. Furthermore, the dose–response correlation between dietary niacin intake and depression was also described.

## Materials and methods

### Study population

This cross-sectional study analyzed data from the NHANES 2007–2016, performed by the Centers for Disease Control and Prevention. The NHANES is a series of cross-sectional, stratified, multi-stage probability surveys for the representative non-institutionalized US civilians to evaluate the health and nutritional status of children and adults. NHANES collects a variety of health-related data, including demographics, diet, physical examination, and laboratory tests. All NHANES study protocols were approved by the ethical review committee of the National Centre for Health Statistics. Also, NHANES obtained the written informed consent of all participants. Methodological details and survey design of the NHANES are publicly available at https://www.cdc.gov/nchs/nhanes/index.htm.

**Fig. 1:**
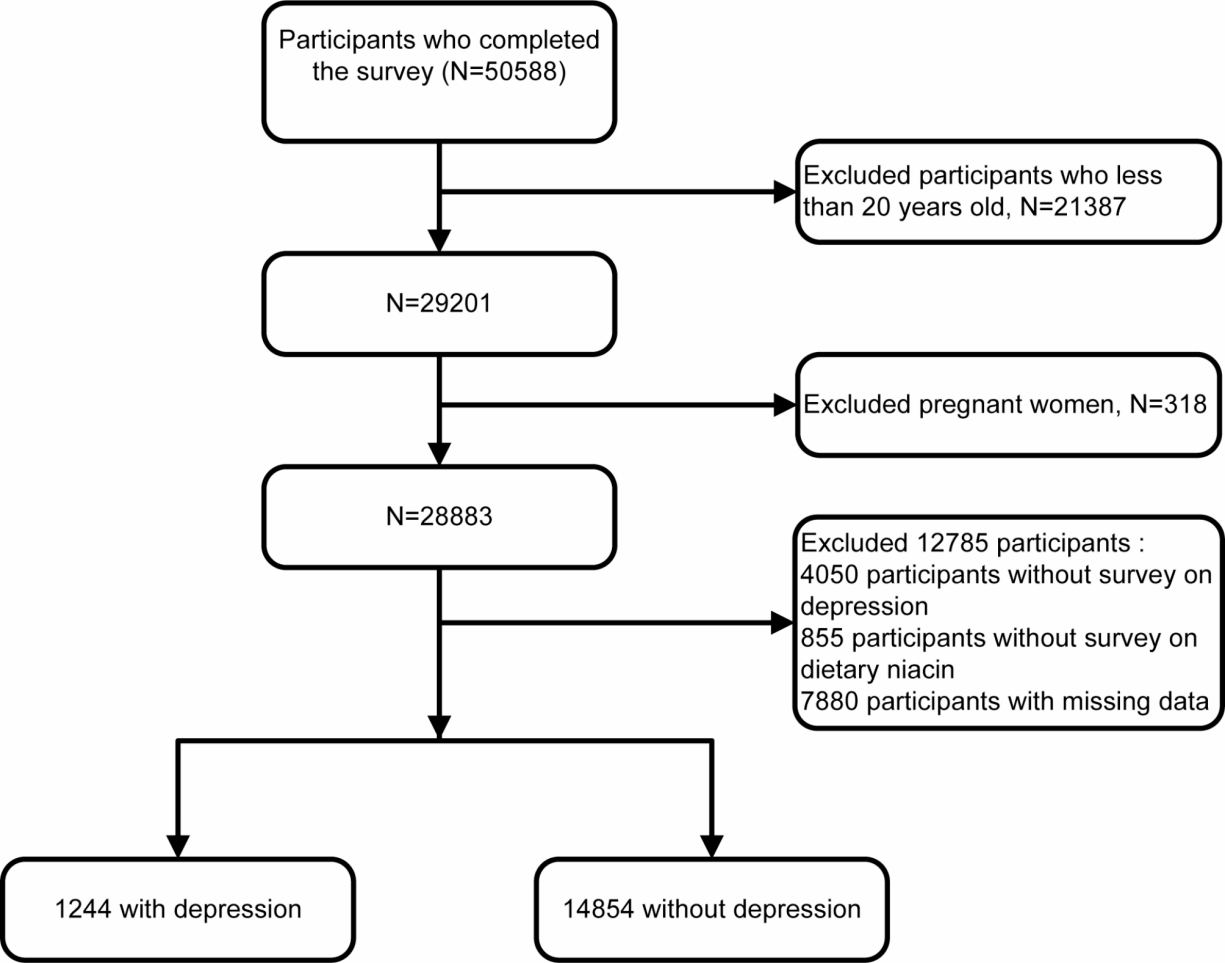
Inclusion and exclusion flow chart

To ensure a high degree of consistency across survey variables and to ensure adequate study power, we combined data from NHANES 2007–2016 into our analyses, which obtained 50,588 participants, and we restricted our analyses to adults aged 20 years and older. Pregnant women and participants who lacked data on depression, dietary niacin intake, covariates, or weight were excluded (Fig. 1).

### Depression classification

In NHANES, the DSM-V-based nine-item Patient Health Questionnaire (PHQ-9) was used to assess depression, a reliable and valid diagnostic tool for examining depression in clinical work and related research settings [13, 14]. DSM-IV based symptom criteria for the nine-item instrument “not at all,“ “several days,“ “more than half the days,“ and “nearly every day” were given a score from 0 to 3, respectively. The scores of each participant were summed to obtain a total score ranging from 0 to 27. Consistent with previous studies, this study defined depression as a PHQ-9 total score ≥ 10, a threshold that has been clinically verified with a sensitivity of 88% and specificity of 88% and was commonly used to define depression in clinical and epidemiological studies [15–17].

### Dietary niacin assessment

Data on dietary niacin intake were collected through 24-hour recall survey. The survey is a retrospective dietary assessment method that provided detailed information on all dietary and beverage consumption in 24-hour period. For each individual, the intake of nutrients from each food or beverage, including niacin intake, were calculated via the US Department of Agriculture’s Food and Nutrient Database for Dietary Studies [18]. Detailed dietary interview methods can be found in the NHANES dietary interview procedure manual [19]. In keeping with previous literature, we used the dietary day-one sample weight for weighting, so the first 24-hour dietary data was included in this study for analysis [20–22].

### Potential covariates

Various potential covariates were included based on the published literature [23–26]. The present study included the following demographic covariates: age (20–44, 45–59, or ≥ 60 years), sex, race, education level (< high school, high school, >high school), marital status (married, living alone, never married) and family income. The poverty income ratio (PIR) divided family income into three categories: low (PIR ≤ 1.3), medium (PIR > 1.3 ~ 3.5) and high (PIR > 3.5). Data on total energy intake were obtained from 24-hour dietary recall. Body mass index (BMI) was categorized as less than 25, 25 to 30, and more than 30 kg/m^2^. According to previous descriptions in the literature, drinking status was classified as never, current and former. And smoking status was categorized as “never” (lifetime < 100 cigarettes), “former” (previous history of smoking but no longer smoking at the time of the interview), or “now” (lifetime cigarette use). Physical activity (PA) can be calculated based on the metabolic equivalent (MET) value, activity type, weekly frequency, and duration [27]. We calculated the value of PA according to the following formula: PA (MET-h/wk) = MET × weekly frequency × duration of each physical activity. PA was divided into three categories: no (MET ≤ 1), low (MET > 1 ~ 48), high (MET ≥ 48). History of hypertension or diabetes was evaluated as self-reported physician diagnosis of hypertension or diabetes.

### Statistical analysis

In accordance with the requirements of the National Centre for Health Statistics, we weighted the data in our analysis. In this study, we combined five 2-year survey cycles of the consecutive NHANES (2007–2016). A specific 10-year dietary weight was created by taking one-fifth of the 2-year dietary weights according to the analytical guidelines available on the NHANES website [28]. Continuous variables were reported by sample-weighted means (standard error, SE), while categorical variables were described as sample-weighted percentages and frequencies. To compare the differences between depression group and no depression group, t-tests (continuous variables) and Chi square tests (categorical variables) were performed. Multiple logistic regression models were used to estimate odd ratios (ORs) with 95% confidence intervals (CIs) of depression at different quartile of dietary niacin. Model 1 was adjusted for total energy intake. Model 2 was additionally adjusted for age and sex. Model 3 was adjusted for age, sex, race, marital status, education, family income, smoking, drinking, physical activity, BMI, hypertension, diabetes, energy, n-3 fatty acids, n-6 fatty acids, folate and zinc.

. We also conducted subgroup analyses to explore whether there were other confounding factors that might influence the association between niacin intake and depression. To assess the robustness of the results of our analysis, we excluded participants with extreme energy intake (consuming < 500 or > 5000 kcal per day) for the sensitivity analysis. Furthermore, restricted cubic spline (RCS) regression was used to investigate the nonlinear relationship between dietary niacin intake and depression. After adjusting for all potential confounding variables, we used a two-piece-wise logistic regression model with smoothing to explore the threshold for the association between dietary niacin intake and depression. Inflection points were identified by using likelihood-ratio test and the bootstrap resampling method. All statistical analysis was conducted by using R software (version 4.2.1). Two-sided p values < 0.05 was considered statistically significant difference.

## Results

### Baseline characteristic

The basic characteristics of the included and excluded individuals are exhibited in the Additional file 1 (Table S1). The baseline characteristics of the 16,098 included individuals are described in Table 1. A total of 1244 individuals with depression, while 14,854 individuals without depression. Compared to the group of participants without depression, those with depression were more likely to be younger, had a higher prevalence of female and tended to be hypertensive, diabetic, living alone. Participants with depression had significantly lower education level, lower family income, lower energy intakes, lower physical activity, were current smokers, were more likely to be former drinkers, had a higher BMI. The daily niacin, n-3 fatty acids, n-6 fatty acids, folate and zinc intake in participants with depression were significantly lower than those without depression. In addition, as shown in the Additional file 2, niacin intake is high, intake of these nutrients (n-3 fatty acids, n-6 fatty acids, folate and zinc) is also high (Table S2).

**Table 1:** Baseline characteristics of study population

	Non-depression	Depression	*P* value
Number of subjects (%)	14,854(92.27%)	1244(7.72%)	
Age (year)			< 0.001
20–44	6977(48.56)	589(49.58)	
45–59	3649(28.78)	371(34.93)	
≥ 60	4228(22.66)	284(15.49)	
Sex (%)			< 0.0001
Male	8097(52.97)	483(40.81)	
Female	6757(47.03)	761(59.19)	
Race (%)			< 0.001
Non-Hispanic White	6886(70.91)	546(64.66)	
Non-Hispanic Black	2962(9.86)	267(12.78)	
Mexican American	2058(7.57)	174(7.78)	
Other Hispanic	1400(4.75)	165(7.93)	
Other race	1548(6.91)	92(6.84)	
Marital status (%)			< 0.0001
Married	9088(63.70)	548(45.45)	
Living alone	2794 (16.02)	387(27.77)	
Never married	2972(20.28)	309(26.77)	
Education (%)			< 0.0001
<High school	1063(3.41)	130(5.64)	
High school	5069(29.82)	571(42.21)	
>High school	8722(66.77)	543(52.15)	
Family income (%)			< 0.0001
Low	4207(19.15)	670(42.60)	
Medium	5494(33.59)	387(33.46)	
High	5153(47.26)	187(23.93)	
Smoking (%)			< 0.0001
Never	8350(56.13)	487(38.55)	
Current	2906(18.71)	499(41.34)	
Former	3598(25.16)	258(20.11)	
Drinking (%)			< 0.0001
Never	1813(9.60)	137(8.26)	
Current	10,809(78.12)	847(73.30)	
Former	2232(12.28)	260(18.44)	
Physical activity (%)			0.03
No	80(0.54)	15(0.96)	
Low	8512(57.06)	750(61.55)	
High	6262(42.41)	479(37.49)	
Body mass index (%)			< 0.0001
< 25 kg/m^2^	4568(32.23)	326(27.79)	
25 to < 30 kg/m^2^	5073(34.58)	331(28.22)	
≥ 30 kg/m^2^	5213(33.19)	587(44.00)	
Diabetes (%)	1466(7.07)	191(11.98)	< 0.0001
Hypertension (%)	4688(27.97)	530(38.07)	< 0.0001
Total energy (kcal) mean (SE)	2231.78(12.13)	2099.67(42.53)	0.035
Niacin (mg) mean (SE)	26.99(0.18)	25.14(0.76)	0.02
n-3 fatty acids (mg) mean (SE)	104.41(3.68)	81.40(16.40)	0.01
n-6 fatty acids (g) mean (SE)	17.26(0.14)	15.87(0.45)	0.004
Folate (ug) mean (SE)	558.06 (4.95)	497.15 (15.15)	< 0.001
Zinc(mg) mean (SE)	12.10 (0.10)	10.49 (0.29)	< 0.0001

### Relationship between dietary niacin intake and depression

After adjustment for all potential covariates, relationship between dietary niacin intake and depression are illustrated in Table 2. Compared to participants in the lowest quartile (Q1) of dietary niacin intake (≤ 15.96 mg/day), the adjusted ORs for depression in Q2 (15.97–22.86 mg/day), Q3 (22.87–32.28 mg/day), and Q4 (≥ 32.29 mg/day) were 0.92 (95% CI: 0.70–1.20, p = 0.51), 0.76 (95% CI: 0.56–0.99, p = 0.04), and 0.68 (95% CI: 0.48–0.98, p = 0.03), respectively. Further excluding individuals with extreme energy intake, 15,757 individuals remained, and the relationship between dietary niacin intake and depression remained stable (Table 3). Compared with participants with lowest niacin intake Q1 (≤ 15.97 mg/day), the adjusted OR values for dietary niacin intake and depression in Q2 (15.98–22.70 mg/day), Q3 (22.71–31.86 mg/day),and Q4 (≥ 31.87 mg/day) were 0.90 (95% CI: 0.69–1.19, p = 0.48), 0.77 (95% CI: 0.56–1.04, p = 0.09), and 0.65 (95% CI: 0.44–0.97, p = 0.04), respectively.

**Table 2:** Association between dietary niacin intake and depression

	OR (95% CI)						
Quartile	No.	Model 1	*P* value	Model 2	*P* value	Model 3	*P* value
**Niacin intake (mg/day)**							
Q1(≤15.96)	4025	1.00 (reference)		1.00 (reference)		1.00 (reference)	
Q2(15.97–22.86)	4026	0.80(0.63,1.02)	0.07	0.81(0.64,1.03)	0.08	0.92(0.70,1.20)	0.51
Q3(22.87–32.28)	4022	0.59(0.45,0.76)	< 0.001	0.61(0.47,0.80)	< 0.001	0.76(0.56,0.99)	0.04
Q4(≥ 32.29)	4025	0.49(0.35,0.69)	< 0.0001	0.54(0.39,0.75)	< 0.001	0.68(0.48,0.98)	0.03
*P* for trend	-	< 0.0001	-	< 0.001	-	0.03	-

Model 1 was adjusted for energy

Model 2 was additionally adjusted for age and sex

Model 3 was adjusted for age, sex, race, marital status, education, family income, smoking, drinking, physical activity, BMI, hypertension, diabetes, energy, n-3 fatty acids, n-6 fatty acids, folate and zinc

**Table 3:** Association between dietary niacin intake and depression in participants with extreme energy intake was not included

	OR (95% CI)						
Quartile	No.	Model 1	*P* value	Model 2	*P* value	Model 3	*P* value
**Niacin intake (mg/day)**							
Q1(≤15.97)	3940	1.00 (reference)		1.00 (reference)		1.00 (reference)	
Q2(15.98–22.70)	3939	0.79(0.62,1.02)	0.07	0.81(0.63,1.02)	0.08	0.90(0.69,1.19)	0.48
Q3(22.71–31.86)	3939	0.58(0.44,0.76)	< 0.001	0.61(0.46,0.80)	< 0.001	0.77(0.56,1.04)	0.09
Q4(≥ 31.87)	3939	0.46(0.33,0.67)	< 0.0001	0.51(0.36,0.72)	< 0.001	0.65(0.44,0.97)	0.04
*P* for trend	-	< 0.0001	-	< 0.001	-	0.03	-

Model 1 was adjusted for energy

Model 2 was additionally adjusted for age and sex

Model 3 was adjusted for age, sex, race, marital status, education, family income, smoking, drinking, physical activity, BMI, hypertension, diabetes, energy, n-3 fatty acids, n-6 fatty acids, folate and zinc

**Fig. 2:**
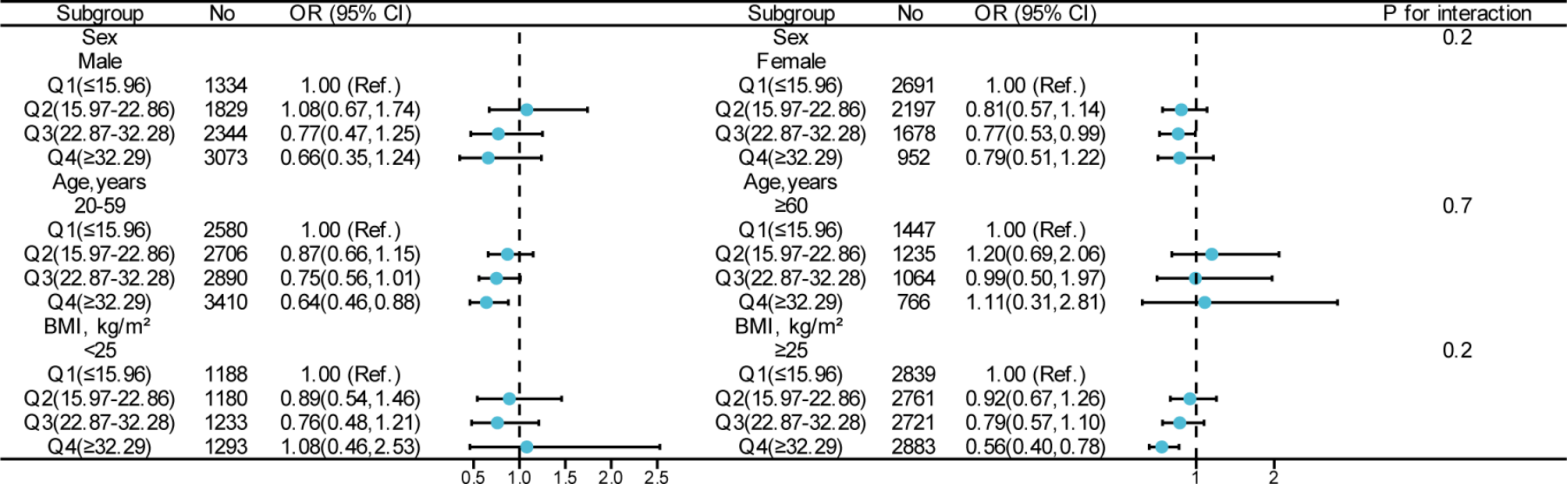
Effect of niacin intake on depression in different subgroup. Except the stratification variables themselves, each stratification factor was adjusted for all other variables (age, sex, race, marital status, education, family income, smoking, drinking, physical activity, BMI, hypertension, diabetes, energy, n-3 fatty acids, n-6 fatty acids, folate and zinc)

Subgroup analysis were performed in several subgroups to evaluate the possible effect modifications of the association between dietary niacin intake and depression (Fig. 2). There were no significant multiplicative interactions (effect modification) for any of these subgroup characteristics in the relationship between niacin intake and depression. Although the negative correlation between dietary niacin consumption and depression seemed to be stronger among female and among participants with 20–59 years old, BMI ≥ 25 kg/m^2^, the interactions were not statistically significant (p for interaction = 0.20, 0.7 and 0.20, respectively).

**Fig. 3:**
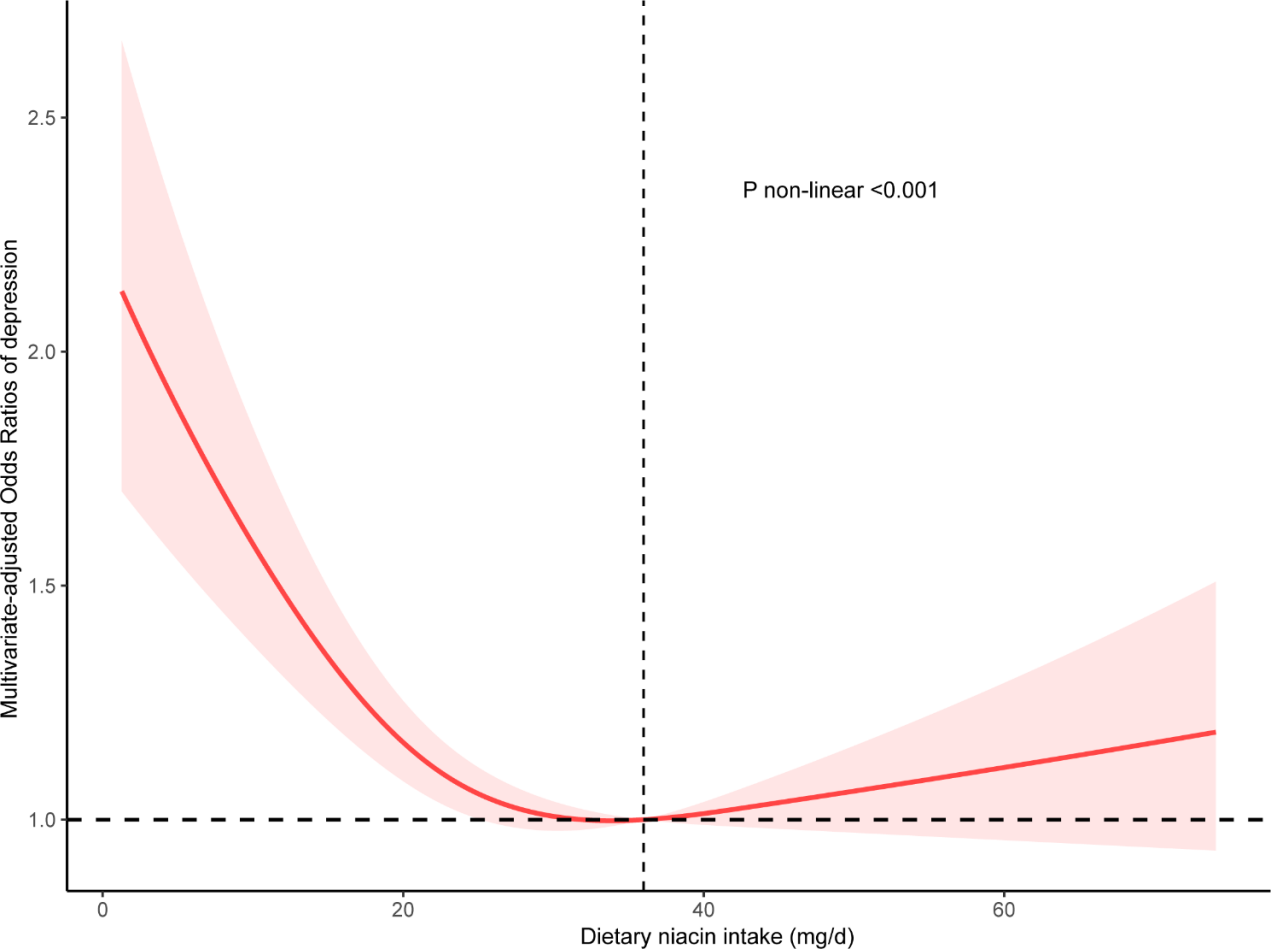
Association between dietary niacin intake and depression odds ratio. The model was adjusted for age, sex, race, marital status, education, family income, smoking, drinking, physical activity, BMI, hypertension, diabetes, energy, n-3 fatty acids, n-6 fatty acids, folate and zinc

In the Fig. 3, the restricted cubic spline showed that a U-shaped correlation between dietary niacin intake and depression (P < 0.001). When niacin intake ≤ 36 mg/day, increased niacin intake was related to a significantly lower risk of depression. When niacin intake > 36 mg/day, the risk of depression elevated with an increase in niacin intake. In the threshold analysis, the OR for depression was 0.98 (95% CI: 0.96–0.99, p = 0.005) in participants with a niacin intake of ≤ 36 mg/day (Table 4). It means that the risk of depression is decreased by 2% with every 1 mg increase in daily dietary niacin intake. There was positive relationship between niacin intake and depression when the daily niacin intake was > 36 mg/day (Table 4). It means that depression incidence rose with increasing dietary niacin intake. According to these analysis, 36 mg/day of niacin intake was observed to be the optimal consumption of niacin in relation to depression incidence.

**Table 4:** Threshold effect analysis of the association of niacin intake with depression

Niacin Intake mg/day	Adjust OR (95% CI)	*p*-value
≤ 36	0.98(0.96,0.99)	0.005
> 36	1.04(1.01, 1.06)	0.03
Log-likelihood ratio test		< 0.001

Fully adjusted for age, sex, race, marital status, education, family income, smoking, drinking, physical activity, BMI, hypertension, diabetes, energy, n-3 fatty acids, n-6 fatty acids, folate and zinc

## Discussion

In this nationally representative cross-sectional study, we found a negative relationship between dietary niacin intake and depression. The sensitivity analyses indicated a robust relationship between dietary niacin intake and depression in American adults. The results were not modified by sex, by age and by BMI. And the relationship between dietary niacin intake and depression exhibited a U-shaped curve (nonlinear, p < 0.001). Moreover, the dietary consumption of niacin to minimize the risk of depression was around 36 mg/day.

Although existing study on the correlation is still rare, the use of niacin to intervene in the clinical treatment of depression has long been a hot topic of study. Back in the 1950s, niacin was used to treat schizophrenia manic depression, benign depression and tension anxiety conditions, and both depressive and anxiety reactions were alleviated with niacin supplementation [29]. A recent study showed that aqueous niacin skin flushing treatment significantly improved depression, anxiety and somatic symptoms in 30 patients with recurrent monophasic depression [30]. It is noteworthy that all of the researches above are case reports or case series, and no additional study has been undertaken to explore the relationship between dietary niacin intake and depression in the general population. The NHANES affords us the unique chance to evaluate whether there is a relationship between dietary niacin intake and depression. which will provide a basis for further determination of the efficacy of niacin in depression.

The association between dietary niacin intake and depression was U-shaped. depression risk was lowest when dietary intake of niacin was around 36 mg/day. Several possible explanations may contribute to a better understanding of this non-linear relationship between dietary niacin consumption and depression. One of the more plausible explanations is the intake of other nutrients that could counteract the effect of dietary niacin intake on depression. The American diet is characterized by a richness in animal protein, refined carbohydrates, and an elevated proportion of n-6 and n-3 polyunsaturated fatty acids (PUFAs) [31]. In fact, n-6 PUFA intake gradually increased with the increment in animal protein (such as fish) consumption. Also, a previous study showed that n-6: n-3 ratio was positively associated with the prevalence of depression [32]. Another alternative explanation for this result is the existence of a threshold effect, whereby once the threshold is achieved, the negative relationship with subsequent higher niacin intake tends to change. Finally, we cannot rule out the possibility of reverse causality. Some participants with depressive symptoms (and possibly subclinical depression) may increase their niacin intake to improve their symptoms.

In the present study, it is worth noting that the negative correlation between dietary niacin intake and depression seemed to be stronger among female and among participants with 20–59 years old, BMI ≥ 25 kg/m^2^. Dietary intake is an important source of nutrients for the body, which is absorbed by the body in the small intestine. foods rich in niacin included fish, meat, milk, peanuts, and enriched flour products [7]. The body consumes these foods along with an increased intake of other nutrients, such as folate [33]. And our findings also showed that niacin intake is high, intake of folate is also high. Women, especially those of childbearing age, are becoming more aware of folate supplementation and are actively taking appropriate amounts of folate before and during pregnancy, and dietary folate intake is the preferred method of supplementation [34–36]. Thus, dietary intake of folate in women may enhance the effect of dietary niacin intake on depression. It is now mostly believed that the gastrointestinal tract is less functional in the elderly than in younger people [37]. As a result, older adults may have a reduced intake of dietary niacin. Therefore, niacin absorption may be high in individuals aged 20–59 years and in women, which may be the reason why dietary niacin was associated with depression only in individuals aged 20–59 years and in women. Current research suggests that the association between being underweight or overweight and depression is controversial [38]. Future studies are required to validate these results. In sum, the negative association between niacin intake and depression among individuals with female, 20–59 years old and BMI ≥ 25 kg/m^2^ was important for proposing individualized strategies to prevent depression in adults. Thus, we recommend that those adults should consume an appropriate amount of niacin rich foods.

Although the underlying mechanism of the negative relationship between niacin intake and depression is still to be explored, our results are biologically plausible based on the available evidence. Several studies have indicated that patients with depression have higher levels of oxidative stress and inflammation, as well as a relatively low intake of dietary antioxidants [39, 40]. For individuals with depression, it is important to have a food source of antioxidants. Niacin decreases oxidative stress in endothelial cells by raising NADP level, reducing glutathione and suppressing the production of reactive oxygen species [41]. Niacin, when consumed in moderation, increases serum 5-Hydroxytryptamine levels, improves brain energy deficits and has powerful antioxidant properties, which may be a biological mechanism for increasing niacin intake to prevent depression. Furthermore, niacin may influence the conversion of macrophages from M1 (pro-inflammatory) to M2 (anti-inflammatory) via the niacin receptor GPR109A, promoting an anti-inflammatory process and thereby inhibiting the inflammatory response [42]. Niacin also reduces the secretion of pro-inflammatory mediators, such as TNF-α, IL-6 and MCP-1, thereby decreasing the body’s inflammatory response [43]. However, more prospective studies are needed to verify the preventive effects of niacin on depression.

There are some limitations that must be considered in present study. First of all, dietary information was obtained from one-time recall surveys, which may not accurately reflect an individual’s usual diet and the total amount of niacin in the body. However, some studies have shown that 24-hour recalls of daily dietary intake may be sufficient for evaluation [44, 45]. Secondly, since the study was conducted on US adults and did not include special groups such as minors, we cannot analyze special populations or other ethnicities due to the limited sample size. Therefore, further researches are necessary to verify the generalizability of these results. Third, the effect of nonrandom missing data on the results are unable to be excluded due to differences in baseline between included and excluded individuals. Furthermore, we cannot eliminate the possibility that the observed relationships are due to unmeasured and unincluded confounders, although we have adjusted for various confounding variables. Finally, our study was a cross-sectional study, which meant that causal inferences cannot be made. Therefore, we need to perform the large cohort study to obtain further accurate evidence. This study presented several advantages. First, this study provided epidemiological evidence of the significant association between dietary niacin intake on depression in a representative general population across the United States. Second, with the adjustments for potential confounding factors, our conclusions are more realistic. And, stratified analyses were performed in several subgroups to identify any existing differences. Furthermore, we evaluated the dose-response effect of dietary niacin on depression and provided practical recommendations. Third, studies on the negative association between niacin intake on depression are relatively rare. This study indicated that moderate dietary niacin intake may help prevent depression.

## Conclusion

In present study, we found that moderate intake of niacin (U-shaped relationship) may protect against depression. Moreover, depression risk was lowest when dietary consumption of niacin was around 36 mg/day.

### Electronic supplementary material

Below is the link to the electronic supplementary material.

Supplementary Material Files 1: Table S1 Basic characteristics of excluded and included participants

Supplementary Material Files 2: Table S2 Population characteristics by categories of dietary niacin intake

## Data Availability

This study analyses publicly available data. The raw data can be found in the repository here: https://www.cdc.gov/nchs/nhanes/index.htm.
