# Supplementary material for: Dietary niacin intake in relation to depression among adults: a population-based study
Source: BMC Psychiatry. 2023 Sep 18;23:678. doi: 10.1186/s12888-023-05188-8 (PMC10506255; doi:10.1186/s12888-023-05188-8)
Supplement: Supplementary file 1 — Supplementary Material Files 1: Table S1 Basic characteristics of excluded and included participants [file 12888_2023_5188_MOESM1_ESM.docx]

**Dietary niacin intake** **in relation to depression among adults: a population-based study**

Sheng Tian, Lanxiang Wu, Heqing Zheng, Xianhui Zhong, Mingxu Liu, Xinping Yu and Wei Wu*

*** Correspondence:** [13807038803@163.com](mailto:13807038803@163.com)

**Table S1** Basic characteristics of excluded and included participants

|  | **Excluded population** | **Included population** | ***P* value** |
| --- | --- | --- | --- |
| Number of subjects (%) | 34490(68.2%) | 16098(31.8%) |  |
| Sex (%) |  |  | <0.001 |
| Male | 16492 (47.8) | 8580 (53.3) |  |
| Female | 17998 (52.2) | 7518 (46.7) |  |
| Age(year) |  |  | <0.001 |
| 20-44 | 4807 (36.7) | 7566 (47.0) |  |
| 45-59 | 3048 (23.3) | 4020 (25.0) |  |
| ≥60 | 5248 (40.1) | 4512 (28.0) |  |
| Race (%) |  |  | <0.001 |
| Non-Hispanic White | 10816 (31.4) | 7432 (46.2) |  |
| Non-Hispanic Black | 8018 (23.2) | 3229 (20.1) |  |
| Mexican American | 7315 (21.2) | 2232 (13.9) |  |
| Other Hispanic | 4113 (11.9) | 1565 (9.7) |  |
| Other race | 4228 (12.3) | 1640 (10.2) |  |
| Marital status (%) |  |  | <0.001 |
| Married | 7496 (57.3) | 9636 (59.9) |  |
| Living alone | 3428 (26.2) | 3181 (19.8) |  |
| Never married | 2158 (16.5) | 3281 (20.4) |  |
| Education (%) |  |  | <0.001 |
| <High school | 2082 (15.9) | 1193 (7.4) |  |
| High school | 5260 (40.3) | 5640 (35.0) |  |
| >High school | 5722 (43.8) | 9265 (57.6) |  |
| Family income (%) |  |  | <0.001 |
| Low | 13210 (44.1) | 4877 (30.3) |  |
| Medium | 10425 (34.8) | 5881 (36.5) |  |
| High | 6288 (21.0) | 5340 (33.2) |  |
| Smoking (%) |  |  | <0.001 |
| Never | 7917 (57.8) | 8837 (54.9) |  |
| Current | 2703 (19.7) | 3405 (21.2) |  |
| Former | 3074 (22.4) | 3856 (24.0) |  |
| Drinking (%) |  |  | <0.001 |
| Never | 2216 (22.1) | 1950 (12.1) |  |
| Current | 5618 (56.1) | 11656 (72.4) |  |
| Former | 2186 (21.8) | 2492 (15.5) |  |
| Physical activity (%) |  |  | 0.403 |
| No | 50 (0.5) | 95 (0.6) |  |
| Low | 6089 (57.9) | 9262 (57.5) |  |
| High | 4372 (41.6) | 6741 (41.9) |  |
| Body mass index (%) |  |  | <0.001 |
| < 25 kg/m^2^ | 17341 (60.7) | 4894 (30.4) |  |
| 25 to < 30 kg/m^2^ | 5431 (19.0) | 5404 (33.6) |  |
| ≥ 30 kg/m^2^ | 5816 (20.3) | 5800 (36.0) |  |
| Diabetes (%) | 2160 (6.7) | 1657 (10.3) | <0.001 |
| Hypertension (%) | 5440 (33.5) | 5218 (32.4) | 0.049 |
| Total energy (kcal)  mean (SD) | 1834.93 (899.79) | 2177.32 (1010.17) | <0.001 |
| Niacin (mg) mean (SD) | 20.77 (13.13) | 26.25 (16.04) | <0.001 |
| n-3 fatty acids(mg) mean (SD) | 101.23(2.34) | 86.58(12.49) | <0.001 |
| n-6 fatty acids(g) mean (SD) | 14.72(0.11) | 12.89(0.23) | <0.001 |
| Zinc (mg) mean (SD) | 10.21(0.08) | 12.39(0.33) | <0.001 |
| Folate (ug) mean (SD) | 548.87(3.97) | 509.28(12.43) | <0.001 |
| Depression (%) | 1190 (11.5) | 1244 (7.7) | <0.001 |

mean and percentages are unweighted.

p value was calculated by independent t-test for continuous variable and Chi-square test for categorical variables.
