# Supplementary material for: Dietary niacin intake in relation to depression among adults: a population-based study
Source: BMC Psychiatry. 2023 Sep 18;23:678. doi: 10.1186/s12888-023-05188-8 (PMC10506255; doi:10.1186/s12888-023-05188-8)
Supplement: Supplementary file 2 — Supplementary Material Files 2: Table S2 Population characteristics by categories of dietary niacin intake [file 12888_2023_5188_MOESM2_ESM.docx]

**Dietary niacin intake** **in relation to depression among adults: a population-based study**

Sheng Tian, Lanxiang Wu, Heqing Zheng, Xianhui Zhong, Mingxu Liu, Xinping Yu and Wei Wu*

*** Correspondence:** [13807038803@163.com](mailto:13807038803@163.com)

**Table S2** Population characteristics by categories of dietary niacin intake

| **Characteristic ^a^** | **Niacin intake, mg/d** | | | | | |
| --- | --- | --- | --- | --- | --- | --- |
|  |  | **Q1** | **Q2** | **Q3** | **Q4** |  |
|  | **Total** | ≤15.96 | 15.97–22.86 | 22.87–32.28 | ≥32.29 | P value ^b^ |
| **No.** | 16098 | 4025 | 4026 | 4022 | 4025 |  |
| n-3 fatty acids intake (mg/day) | 102.78(3.72) | 61.74(3.97) | 79.45(5.13) | 102.90(7.04) | 160.86(8.49) | < 0.0001 |
| n-6 fatty acids intake(g/day) | 17.17(0.14) | 10.94(0.20) | 15.33(0.21) | 18.11(0.22) | 23.40(0.32) | <0.0001 |
| Folate (ug/day) | 553.75(4.49) | 307.04(4.53) | 455.05(5.45) | 577.71(6.73) | 839.12(11.02) | < 0.0001 |
| Zinc(mg/day) | 11.98(0.09) | 6.70(0.09) | 10.12(0.17) | 12.53(0.12) | 17.82(0.21) | < 0.0001 |

^a^ mean was weighted

^b^ p value was calculated by weighted one-way analyses of variance for continuous variable
